# Agriculture

**Published:** 1877-06

**Authors:** 


### AGRICULTURE

Pursued with intelligent industry, affords a larger number of high advantages than any other occupation of human life; it strengthens the body, invigorates the mind; and while it refines the sentiments, it purifies the heart, by compelling it to look upward for reliance and help towards Him *who giveth rain and fruitful seasons.* It curbs inordinate ambitions, by yielding a moderate remuneration for toil, while at the same time it imparts a feeling of quiet confidence in the future, from the declaration that while the world stands, *seed time and harvest shall not cease.* The young man brought up to till the soil, begins to feel gradually that the rewards of his toil, are proportioned to his labor, and this imparts by degrees a spirit of self-reliance, which begets independence, and an amount of industrious activities, worth more to that young man, in his after conflicts with the world, than the inheritance of unearned thousands.

### SUMMERING IN THE COUNTRY, which, as done by the masses is an indescribable, an incomprehensible absurdity. Young gentlemen should go afoot in companies, camp out, fish, hunt, swim, sail, row, botanize, or with hammer and satchel study the rocks and read their individual histories from the hours that time began. Mothers and daughters should hie away to some retired farm house, or to some mountain home, and with sun bonnet and plainest calico dresses in shape most convenient for wandering over the hills and far away, or for mounting their ponies and gallop away in gleeful mood for an hour or two every morning, then read awhile and go a berrying or to the dairy or to the kitchen and try their hands at some new receipts in cookery; then after tea go on foot a mile or two away with brothers and beau to a quilting or a singing school or to meet at some neighbor's house for frolic and fun in the abandon of childhood and innocence; these are some of the wise ways of spending a summer in the country as a means of resting the mind, of renovating the brain, and giving new vigor to the whole physical man. But the misfortune is that very few go to the country in summer with such aims and ends. The first and main desire of many is a " good table;" three times a day they want spread before them the richest food, the most enticing viands, the most luxurious delicacies, and if these are not secured, all the advantages of the seaside and the spa fail to avert from the place their unstinted anathemas. If, on the other hand, a retired place in the country is secured, there is nothing to interest them, and the greater part of their waking existence is spent in lounging on a sofa, in lolling in a rocking-chair or in dozy dreaminess on a bed. Once in a while they take a listless stroll through the "grounds" of the establishment, if, indeed, these "grounds" are any thing more than a cow pasture or a weedy "garden" with here and there a morning glory, a hollyhock and a marigold. The morning paper is the greatest event of the day; after that comes "Harper, or Godey, or the Atlantic monthly; but these are too heavy for their light heads, and soon they fall to stretching and yawning, then overpowered by sleep until the gong or steer's horn wakes them up, they feed without relish and without appetite, because they take no exercise, for in the morning the dew is on the grass and they can't walk out, at noon it is too hot, after dinner they are sleepy, and they must have a "nap;" then it is so sultry and dusty that no pleasure can be expected either from a drive or promenade unless it is after tea, and then the night air is the great bugbear. Very soon this inactive, jejune life brings on a loss of appetite, and they expect their host to wake it up by having some new dish; a large part of their time is spent in wondering what will be for the next meal and criticising the last. And thus, after all, spending a summer in the country means an unvarying round of eating lounging, sleeping and listlessness.

This going to the country for the summer is a great need for old and young in the large cities, growing children especially; but the way it is managed generally it is a perfect farce. Just look at the items. You exchange a fine house with large, airy rooms and ample closets for one single apartment, which, by the time you have your trunks arranged, leaves you barely space to turn round; if you want to hang up your coat you must go to the "village" and get a nail. If you desire a drink of water you must go down stairs for it, or more likely, to the kitchen or pump, for in the "parlor" you find that children and nurses have monopolized every "tumbler" and forgot to remove certain remnants of sweet cake from around the edges and the bottom of the glass. If you want a cup of hot water you must go to the cook, and be very polite too, or you won't get it until next day. At nightfall you go groping about with a tallow candle or a kerosene lamp, with its long, tottering chimney, which you are every moment afraid will tilt over, leave you in Egyptian darkness, or blow you, and may be, the whole establishment, sky high, besides spoiling the old woman's carpet. The first morning you come down to breakfast, your mouth fairly waters at visions of sweet butter, fresh eggs and pure milk, but you soon find that the butter might have been sweet a year or two ago, but not now; the very first egg you crack open has a spring chicken in it, but you expected to have spring chickens at a country farm house as a matter of course, and now you have them sooner than you expected; as to the milk, it had been sent for sale to the village the day before, and this remnant was put on the table to prevent its going to waste, and as it was to be mixed with coffee, it made no difference if it did stand around in white spots. The bread! is it the crisp French roll of the city? Nothing of the sort, it is either of a leaden sogginess, or so yellow that it reminds you of an apothecary shop, and you begin to wonder if the entire stock in trade of saleratus of the village druggist was not put into that one plate of biscuits. In a very short time you find that the "variety" of the establishment has run out. You have ham for breakfeast, corned beef for dinner, and sour preserves for supper; then apace come the hot nights; you are in the attic and you fairly sweat in the daytime, and stew and fret and fume in the night, the perspiration streaming at every pore, while the lively musquito keeps up his jubilee; but he soon gets his fill and troubles you never again; at midnight, however, other foes must feed, and you turn and scratch, and scratch and turn until the morning begins to dawn, when exhausted nature falls into the arms of Morpheus as helpless as a baby; but you don't stay fallen thus for a single hour, for the impertinent fly has had nothing to eat all night and is now perfectly ravenous, and in his winding way over face and nose and forehead and lip, searching for a convenient spot to thrust his spear into, he breaks in upon your dreams and rouses you up to the realities of a "summer in the country." Compare such a life with the luxuries of your own city home ; the convenient gas, the iced croton, the hot water always ready at your elbow day or night by the turning of a fawcet, the privilege of roaming from one spacious apartment to another, to the front room in the morning, and rear at night or vice versa, so as to baffle the heating sun ; the spacious hair mattress, springy and cool. At breakfast, the yellow butter, made but yesterday, the luscious cream-cup, the French rolls, and the porter house, with the Times, Herald or Tribune to tell you what was said twelve hours before by Disraeli, or what was done by Bismarck, what Turkey fears, what Russia hopes, and what of progress is making in our own land of promise and of hope. After breakfast you take your walk on dry pavements on the shady side of the street, at midday you keep quiet in the cool of your office or counting-room, and at evening are fanned by the gentle west wind as you take your plate of Fuscell's ice cream at nine o'clock with your friends seated around you, and then retire in the confidence of sweet dreams, and sleep undisturded by insect vampyres, because your wife is housekeeper.

Everybody who possibly can, ought to leave New York for the country sometime during the summer ; but to do so with advantage, the great aim should be recuperation, rest for the brain, rest for the mind and physical repose. To accomplish these, get all the sleep you can during the night, be out in the open air every hour possible during daylight, in occupations which will compel the mind away from its usual routine of engagement in the pursuit of something which interests, instructs or profits. Exercise enough after breakfast in the open air to make you feel so hungry that bread and butter will taste sweet to you ; exercise enough in the open air after a noon-day dinner to digest that dinner most thoroughly ; eat not an atom between meals, and with a piece of cold bread and butter and a cup of any warm drink at sundown, pass the evening in joyous or instructive social intercourse. Spending a summer thus in the country, you will return to the city with a mental activity and a surplusage of general bodily health, which will fit you for another year's work in the great struggle for fame, fortune, or better still, usefulness to your kind.

